# A vision physiological estimation of ultraviolet window marking visibility to birds

**DOI:** 10.7717/peerj.621

**Published:** 2014-10-09

**Authors:** Olle Håstad, Anders Ödeen

**Affiliations:** 1 Department of Anatomy, Physiology and Biochemistry, Swedish University of Agricultural Sciences, Uppsala, Sweden; 2 Department of Animal Ecology, Uppsala University, Norbyvägen, Uppsala, Sweden

**Keywords:** Window collision, Colour vision, Ultraviolet light, Avian vision, Spectrophotometry, Visual physiology

## Abstract

Billions of birds are estimated to be killed in window collisions every year, worldwide. A popular solution to this problem may lie in marking the glass with ultraviolet reflective or absorbing patterns, which the birds, but not humans, would see. Elegant as this remedy may seem at first glance, few of its proponents have taken into consideration how stark the contrasts between ultraviolet and human visible light reflections or transmissions must be to be visible to a bird under natural conditions. Complicating matters is that diurnal birds differ strongly in how their photoreceptors absorb ultraviolet and to a lesser degree blue light. We have used a physiological model of avian colour vision to estimate the chromatic contrasts of ultraviolet markings against a natural scene reflected and transmitted by ordinary window glass. Ultraviolets markings may be clearly visible under a range of lighting conditions, but only to birds with a UVS type of ultraviolet vision, such as many passerines. To bird species with the common VS type of vision, ultraviolet markings should only be visible if they produce almost perfect ultraviolet contrasts and are viewed against a scene with low chromatic variation but high ultraviolet content.

## Introduction

**Figure 1:**
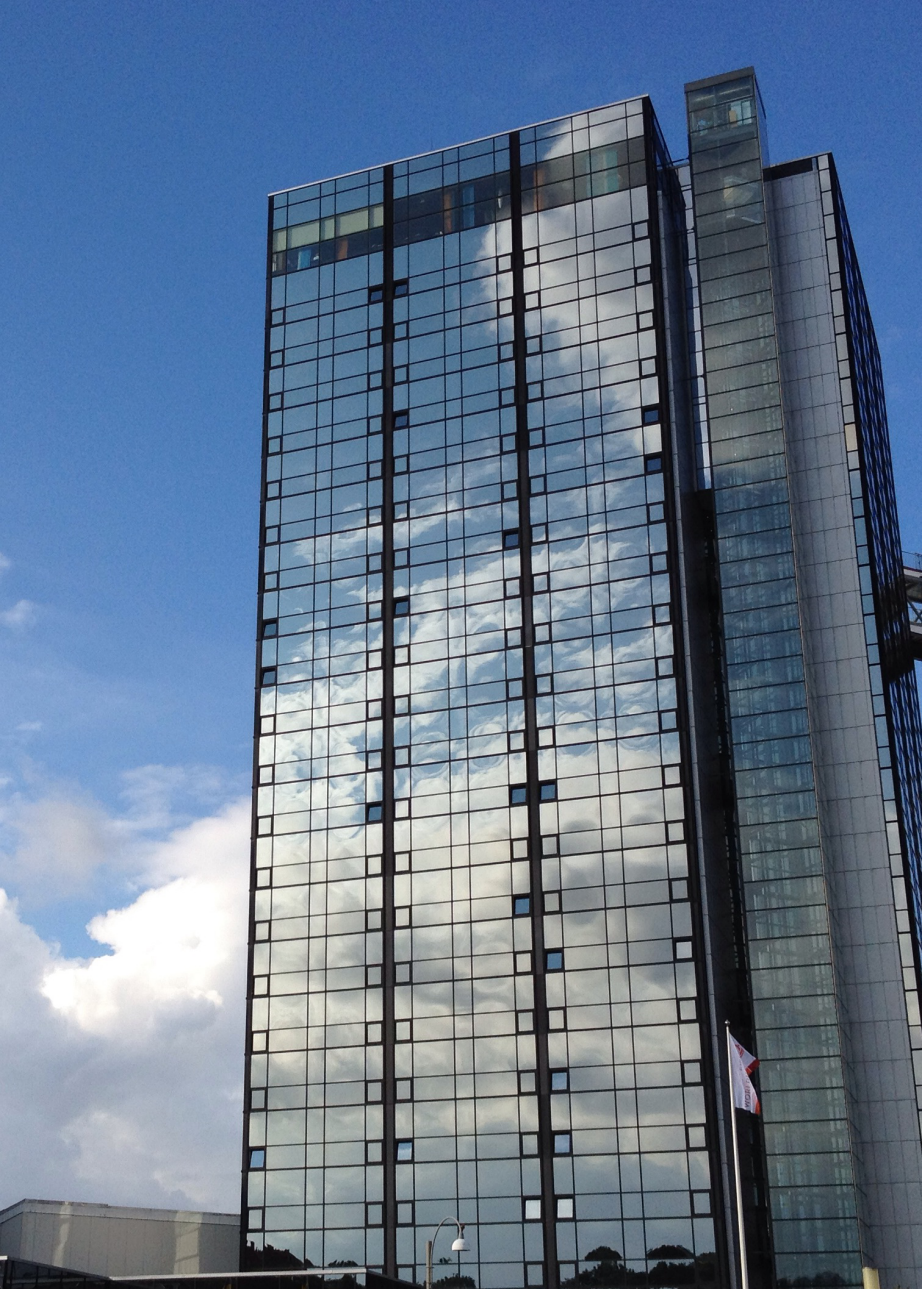
Sky reflected in the windows of a high-rise building in central Gothenburg, Sweden. Photo by Anders Ödeen.

Glass windows cause more bird fatalities than one might think (Banks, 1976). Being optimized for flight, birds are lightly built and collisions with large obstacles often result in serious injury or death. Because the glass reflects the landscape outside (Fig. 1) or offers a more or less unobstructed view of items behind the window as well as the landscape on another side of a building, it may trick birds into believing that the window is an available flight path. As a consequence, the world-wide avian death toll from window collisions reaches billions each year, according to recent estimates (Drewitt & Langston, 2008; Klem Jr, 2009a).

The means to prevent avian window collisions include nets, screens or grilles that are placed at a safe distance in front of windows or densely spaced, visible markings applied to the glass directly. Albeit effective (Rössler, Laube & Weihs, 2007) these solutions diminish the aesthetic value of having window glass in buildings, and will impair the view of the scene outside. Since it was discovered that diurnal birds can see ultraviolet radiation (Huth & Burkhardt, 1972; Wright, 1972) to which humans are blind, reflective or absorbing ultraviolet markings on window glass have been proposed and tested to make birds notice the surface while the marking remains invisible to human observers. However, this seemingly elegant solution to the problem has had varying success (see Haupt, 2011). On the one hand, ultraviolet absorbing stripes on a window with narrow (5–10 cm) spacing have proven almost as effective as covering virtually the whole window with human-visible markings (Klem Jr, 2009b). On the other hand, field tests of commercially available UV-patterned glass have, under see-through conditions, shown an increased likelihood of window collisions compared to ordinary window panes (Klem Jr & Saenger, 2013).

**Figure 2:**
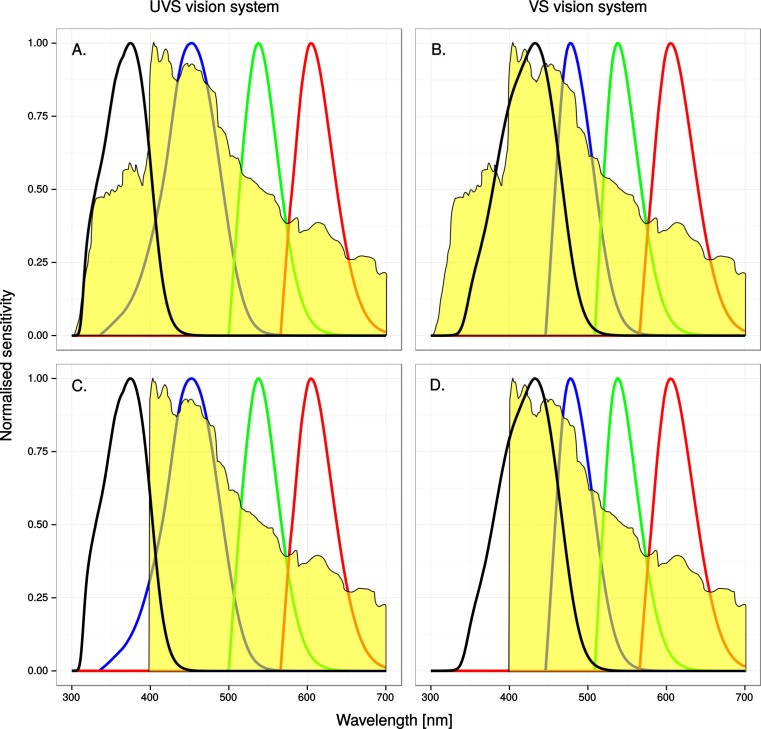
A spectroradiogram of clear sky, unaltered (A) & (B) or modified (C) & (D) to simulate the reflectance of a window marking that completely absorbs ultraviolet radiation below 400 nm, superimposed on normalized single-cone sensitivity. The four curves represent the UV, SWS, MWS and LWS cones, from left to right. See methods for details.

A potentially complicating factor to anti bird-window collision efforts is that birds of different species vary in their sensitivity to ultraviolet radiation. Interspecific variation in the wavelength of maximum absorbance (*λ_max_*) of the class of cone photoreceptors that is responsible for sensitivity in the ultraviolet part of the spectrum falls into two discrete ranges, called UVS (ultraviolet sensitive) and VS (violet sensitive) (reviewed in e.g., Hart & Hunt, 2007). The *λ_max_* in the UVS photopigments range from 355 to 380 nm and in the VS photopigments from 402 to 420 nm, respectively and generally co-varies with other physiological characters in the eye, such as the 445–480 nm *λ_max_* of the SWS (short wave sensitive) cone (reviewed by Hart & Hunt, 2007) and the ultraviolet transmissive properties of the cornea and lens (Lind et al., 2014). Although UVS cone photoreceptors are optimised for ultraviolet sensitivity, most VS birds have a significant degree of ultraviolet vision because the VS cone has a broad sensitivity that reaches into the ultraviolet (see Fig. 2).

Most avian orders and families contain exclusively VS species while parrots, gulls and many passerines are notable examples of UVS species (reviewed by Ödeen & Håstad, 2013). It’s also notable that dual-choice flight tunnel experiments and field tests with ultraviolet markings that have successfully alerted birds to window obstacles in their flight path (Ley, 2006; Klem Jr, 2009b) have almost exclusively involved Passerida passerines, and Passerida passerines are UVS birds with no known exception (e.g., Ödeen, Håstad & Alström, 2011). It is reasonable to assume that the interspecific variation in ultraviolet sensitivity is important to the effectiveness and design strategies of anti-collision markings of windows, but this has very rarely been considered (cf. Rössler, Laube & Weihs, 2007) and never investigated experimentally.

The visibility of any chromatic contrast depends not only on how the observer’s cone sensitivities are distributed across the spectrum but also on the strength of the contrast in comparison to the natural noise levels of the observer’s colour channels (the cones and their associated neural pathways) (Vorobyev & Osorio, 1998). One cannot *a priori* assume that ultraviolet markings will produce detectable chromatic contrasts against natural scenes for animals with a certain capacity of ultraviolet vision. The chromatic signals have to exceed the noise levels set by the retinal density of ultraviolet sensitive photoreceptors to be visible and whether they do so depends on both the absolute and relative intensity of light across the spectrum in the scene (Vorobyev & Osorio, 1998).

We have used data on avian colour vision to estimate the potential effectiveness of simulated ultraviolet window markings as viewed against spectrophotometrically measured backgrounds. The scenes against which the markings were viewed in the simulated models were either reflected in or transmitted through commercially available window glass situated in an actual building. The avian observers were modelled as belonging to UVS and VS species.

## Materials and Methods

We modelled avian perception of four collision prone scenarios: a bird viewing the sky or the natural background of coniferous or deciduous trees, either transmitted through or reflected in a glass window. For this purpose we collected four kinds of spectrophotometric irradiance data: (1) ambient light from a clear sky, (2) directly from clear sky (Fig. 2), cumulus clouds, a pine tree and an apple tree with green leaves and yellow fruit, (3) reflections from a wall mounted triple-glass window (Combiglas Energi, Combiglas AB, Sweden) with dark background, 4 m above ground level, of the same clear sky, cumulus clouds and pine tree and (4) of the same clear sky, cumulus clouds and apple tree but through a triple-glass window (Combiglas Energi). All measurements, except from sky and clouds were taken at a distance of ca. 10 m. We used a calibrated telespectroradiometer, purpose built according to Sumner, Arrese & Partridge (2005). It consisted of an AvaSpec-2048FT-SPU portable spectroradiometer (Avantes, the Netherlands), connected via a UV–VIS, 400 µm fibre-optic cable (Ocean Optics, the Netherlands) to the focal plane of a modified Nikon F801 SLR camera with a UV-Nikkor 105/4.5 quartz lens (Nikon Corporation, Tokyo, Japan) and a purpose built quartz filter. The measured spot was 4 cm in diameter at 10 m from the telespectroradiometer. A Nikon HS-8 s (Nikon Corporation, Tokyo, Japan) lens shade was attached to the lens. The lens aperture was set at f/4.5 and focus set at the object under measurement, i.e., never the window itself. The modified camera was mounted on a 1.51 m high tripod during all measurements. For the ambient light measurements we used a calibrated spectroradiometer, S2000, connected to a cosine corrector (Ocean Optics, the Netherlands) via the same fibre-optic cable. The cosine corrector was fixed to the end of a 2.57 m high pole, with the light-collecting surface aimed horizontally along the azimuth angle of the sun.

We took the ambient light measurements in an open field in Hammarskog, Uppland, Sweden on a clear morning, 26 April, 2007, from 30 min before sunrise over a nearby tree line to 30 min after (equal to 15 min before and 45 min after sunrise over the horizon). All other measurements were taken in a suburban setting in Uppsala with open gardens and 1–2 story houses on 16 August 2013 at 3 pm, a partially cloudy day, aiming the lens of the telespectroradiometer ca. 15°N.

We used the Avantes AvaSoft 7.0 software to record the spectrograms. These were then converted and imported to purpose written software (Håstad & Ödeen, 2008). Each spectrum was analyzed from 300 to 700 nm and interpolated to a step width of 1 nm. Spectra were standardized to counts per millisecond, controlled for the equipment used and transformed to Q/m^2^/s/nm.

Colour distances in the eye of the observer were calculated using the Vorobyev–Osorio noise limited discriminability model (Vorobyev & Osorio, 1998) as defined in Schaefer, Schaefer & Vorobyev (2007). This vision physiological model quantifies how differently two colours should be perceived in units of just noticeable differences (jnd), where a pair of colours with jnd below one are indistinguishable to the modeled eye. One jnd is the eye’s performance threshold at ideal lighting and viewing conditions but the threshold is raised under deteriorating conditions (Osorio et al., 2004). The UVS vision system was modeled using cone sensitivities, including the effects of absorption in the cone oil droplets and the ocular media, of blue tits, *Cyanistes caeruleus* (Hart et al., 2000), and the VS using peafowl, *Pavo cristatus* (Hart, 2002) with cone proportions of 1 (UVS/VS), 1.92 (SWS), 2.68 (MWS: medium wave sensitive cone), and 2.7 (LWS: long wave sensitive) for UVS (Hart et al., 2000) and 1, 1.9, 2.2, and 2.1 for VS (Hart, 2002). The sensitivity of the eye is affected by the amount of available light; quantum flux (Q/photoreceptor/s) according to Vorobyev (2003) was set to 20,000 for daylight light intensity and for pre-dawn conditions to 500 (Osorio et al., 2004).

For a window marking to be detected by a bird we assumed that it not only must be visible, but also deviate from the expected colour variation present in the background (for example, a red dot may be clearly visible against the average colour of an apple tree, but not stand out when viewed against the green leaves and ripe apples). We therefore added a criterion of visibility to the 1 jnd criterion, namely that the mean of pairwise contrast in a window marking must fall outside the confidence intervals of the mean of the background variation. Colour distance matrices of five random spectra from each background type (clear sky, cloud or tree in data type 2–4 above) were calculated using the Vorobyev–Osorio noise limited colour discriminability model. Each matrix thus contained ten colour distances (e.g., between five parts of the clear sky reflected in the window), and is presented in the graphs as a geometric mean of distance values and a 95% confidence interval. Since colour distances can only take values above zero, we log-transformed the data before calculating the means and confidence limits.

**Figure 3:**
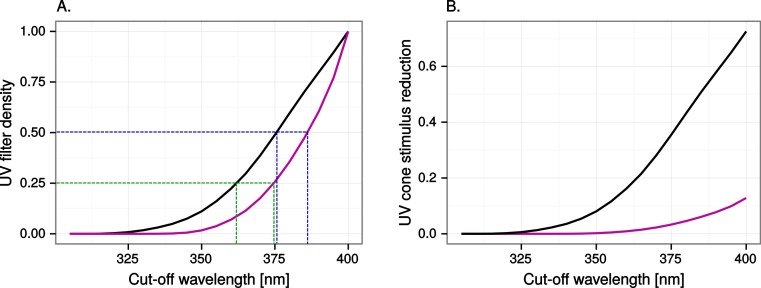
Ultraviolet reduction as a function of cut-off wavelength. (A) Translation between the reductions in ultraviolet modelled in this study (UV filter density) and long-pass cut-off wavelengths normally used in commercial ultraviolet markings. For example, ORNILUX Mikado is claimed to transmit 0% radiation below 380 nm (ORNILUX Bird Protection Glass, 2014; ISO standard UV is <380 nm), which is equivalent to at least 50% reduction of ultraviolet for a UVS bird (black line to the left) and at least 25% for a VS bird (purple line to the right). (B) Reduction in UV cone stimulus resulting from 100% long-pass filtration at various wavelengths.

We simulated ultraviolet markings, window markings with ultraviolet contrast of varying strength, through reducing by 25, 50 or 100% the light at wavelengths below 400 nm in the reflectance and transmittance spectra. We chose 400 nm as a conservative limit. Radiation below this wavelength is almost invisible to humans (Stockman, MacLeod & Johnson, 1993) but visible to birds in general, which have ultraviolet sensitive visual pigments (Ödeen & Håstad, 2013), and ultraviolet transparent cone oil droplets (Hart & Hunt, 2007) and ocular media (Lind et al., 2014). Setting the limit lower in wavelength would have significantly reduced the visibility of ultraviolet markings to the VS vision model but only very marginally affected visibility to human observers. Although commercial applications (e.g., the ORNILUX Mikado bird protection glass; Arnold Glass, Germany) usually reduce ultraviolet by long-pass filtration, we chose to model the ultraviolet markings as density filters in order to keep ultraviolet reductions constant regardless of vision system (VS or UVS). Our ultraviolet reductions can be graphically translated into long-pass cut-off wavelengths with the help of Fig. 3A.

We then calculated distance matrices against the same but unmanipulated spectra. In doing so we assumed that the glass acts as a perfect reflector and transmittor across the wavelength range of interest, 300–700 nm. We also simulated the effects of window markings on the test windows, including their actual reflectance and transmittance. The reason for simulating a perfect glass was to isolate the effects of the ultraviolet manipulations from the optical properties and imperfections of real windows. The tree background was the apple tree in the perfect glass and real window glass transmittance distance matrices and the pine tree in the real window glass reflectance matrices.

In this study we use the term ultraviolet markings to mean any modification of the glass that exclusively changes the relative amount of ultraviolet radiation transmitted or reflected. As a surface may only reflect up to 100% of the incident light, a modification of the surface to increase the ultraviolet reflectance will only be visible if the area surrounding the reflector differs in reflectance, i.e., is ultraviolet absorbing. We therefore did not explicitly model ultraviolet reflective markings as these from the observer’s perspective are equivalent to increasing the surrounding absorbance.

## Results

**Figure 4:**
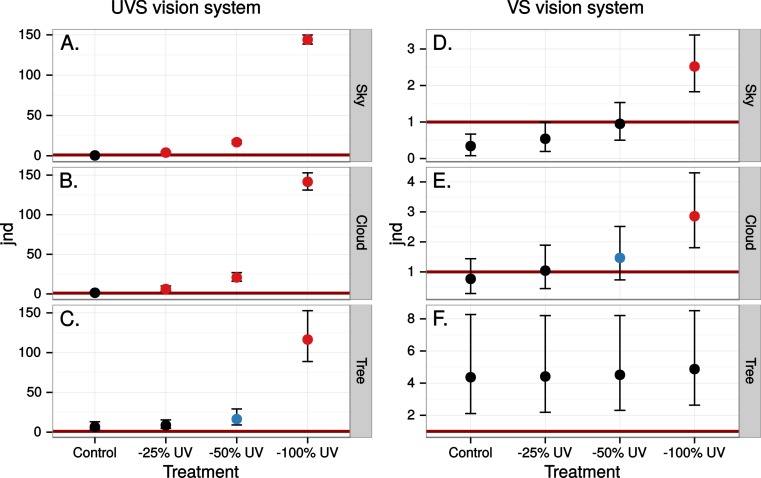
Estimated visibility of simulated markings on perfectly reflecting or transmitting window glass. The simulated markings reduce ultraviolet (UV) by 25, 50 or 100% compared to unmanipulated glass (Control). Visibility is shown in units of just noticeable differences (jnd), i.e., chromatic contrasts between randomly chosen pairs of patches in the scene, one patch viewed through the marking and the other through the clear window, Geometric mean jnd (points) with 95% confidence intervals (bars) are shown in the graphs. Blue colour means that the mean falls outside the confidence interval of the control and is above 1 jnd. Red colour means in addition that the confidence interval is completely outside the confidence interval of the control. Visibility is modelled from birds with UVS (A–C) and VS (D–E) type of colour vision.

The simulated 100% ultraviolet absorbing window marking on the simulated perfect window drastically and selectively reduced the amount of UV cone stimulus (72%) in a UVS model of avian colour vision. However, this effect was only moderate (13%) in the case of VS colour vision (Figs. 2 and 3B). The relative reduction in UV cone stimulation resulted in a sizeable difference between the two vision systems in how strongly colour contrasts between the window marking and the natural background of trees and sky were perceived in the physiological model (Fig. 4). Ultraviolet reductions of 50% made markings clearly visible to a UVS bird. That is, mean chromatic contrasts between the patches measured through simulated markings and the same unmodified patches were well above 1 jnd, and above the 95% confidence limits of the chromatic contrasts resulting from natural variation in the background scene alone. The 25% ultraviolet reductions produced mean chromatic contrasts that were clearly stronger than the confidence intervals of the natural variation of the sky and cloud backgrounds. In the cases of trees however, the reduction of ultraviolet only formed a mean contrast that fell within the confidence limits of the natural background variation. The visibility of the simulated markings was clearly poorer to the VS vision system. Mean chromatic contrasts exceeded 1 jnd in all cases of 100% ultraviolet reduction, and in 50 and 25% of clouds and trees. However only 100% ultraviolet reduction of sky and 100 and 50% of clouds led to means above the confidence limit of the background variation.

**Figure 5:**
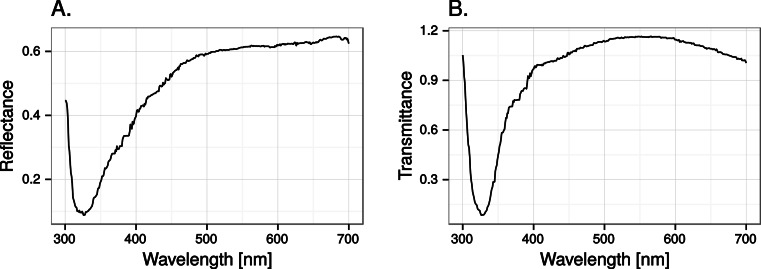
Reflectance (A) and transmittance (B) of cumulus cloud spectra in the test windows.

**Figure 6:**
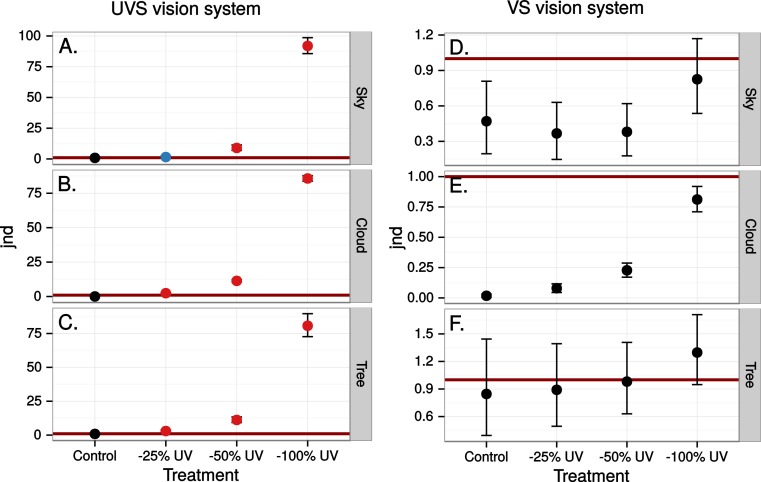
Estimated visibility of simulated ultraviolet markings as in Fig. 4 but in a scene reflected in a real window.

**Figure 7:**
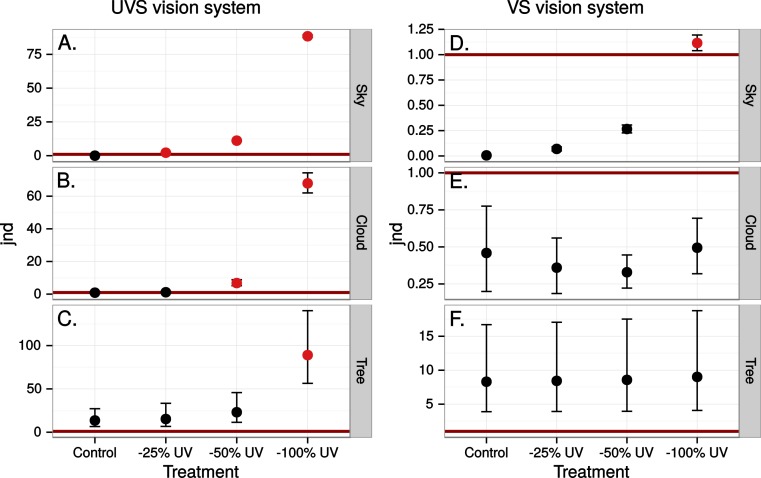
Estimated visibility of simulated ultraviolet markings as in Fig. 4 but in a scene transmitted through a real window.

Close to 50% of light, in the wavelength range visible to humans, was lost when reflected in the test window, while transmittance in the same wavelength range was very high (Fig. 5). In both cases there was a marked attenuation of radiation in the ultraviolet range. All chromatic contrasts were then reduced compared to the perfect reflector/transmittor cases (Figs. 6 and 7). According to our criteria, the UVS system gained visibility of the 25% ultraviolet reducing window marking against the reflection of a tree but lost visibility in the 25% ultraviolet reduction of cloud transmittance. No treatment for the VS-system reached a mean of 1 jnd except the 100% ultraviolet reduction in the reflected tree but the mean did not surpass the confidence limit of the chromatic variation of the background.

**Figure 8:**
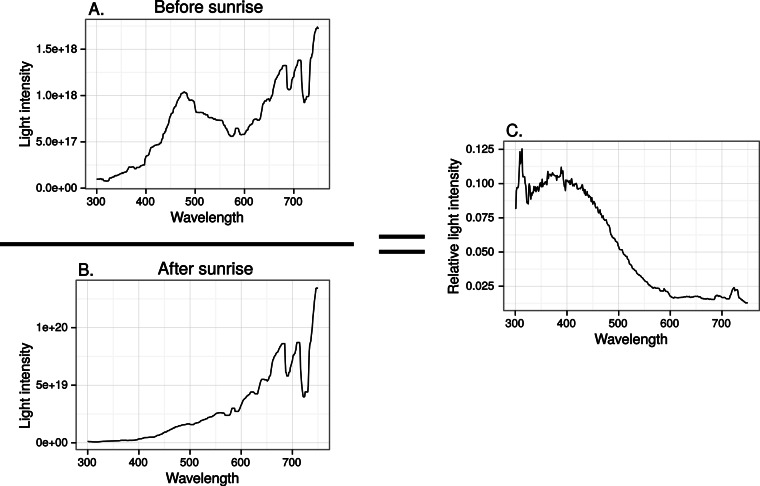
Relative spectral content in the horizontal direction of the sun before (A) and after (B) sunrise in late April, in Uppland, Sweden.

**Figure 9:**
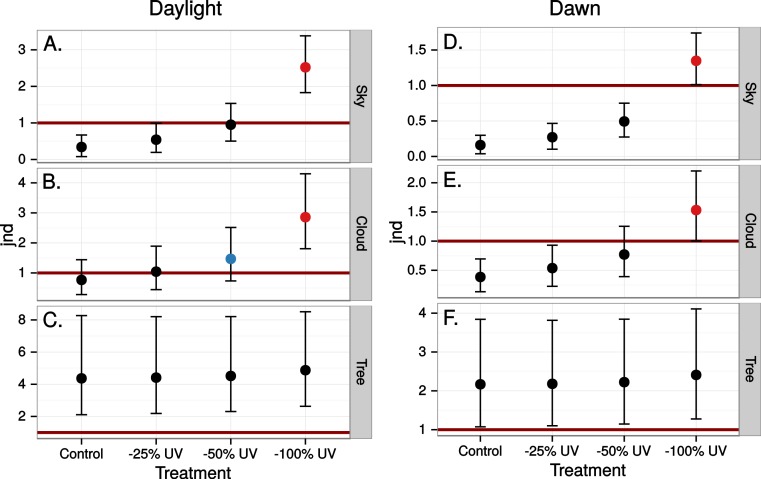
Estimated visibility of simulated ultraviolet markings to the VS bird in Fig. 4, but comparing visibility in the middle of the day (A)–(C) to conditions at dawn (D)–(E).

Finally we estimated the chromatic contrast to a VS bird that simulated what ultraviolet markings would produce on perfectly reflecting window glass but at dawn, when the proportion of UV wavelengths in the ambient light is higher than during mid-day (Fig. 8) but also when the total light intensity is lower (500 vs. 20,000 Q/photoreceptor/s: Vorobyev, 2003). All mean chromatic contrasts against sky and cloud, except the 100% reduction treatments, then fell below 1 jnd (Fig. 9) suggesting that the contrast-increasing effect of stronger relative contribution of ultraviolet is overpowered by increased colour channel noise from the lower overall light levels at dawn compared to mid-day.

## Discussion

At least 50% ultraviolet absorbing (or reflecting) window markings appear to be visible against a natural scene for birds with UVS vision, such as gulls, parrots and Passerida passerines. Birds with VS vision however, such as raptors, ducks and geese, and pigeons, are unlikely to perceive such ultraviolet markings (for a list of UVS and VS species see Ödeen & Håstad, 2013). The low ultraviolet reflectance and transmittance of real windows (e.g., Fig. 5) make ultraviolet markings even less visible to VS birds. Furthermore, there is no improvement of ultraviolet marking visibility under lighting conditions at dawn, even though they are relatively ultraviolet rich compared to daylight conditions. The reason most likely being that low light intensities in the morning reduce overall contrast.

**Figure 10:**
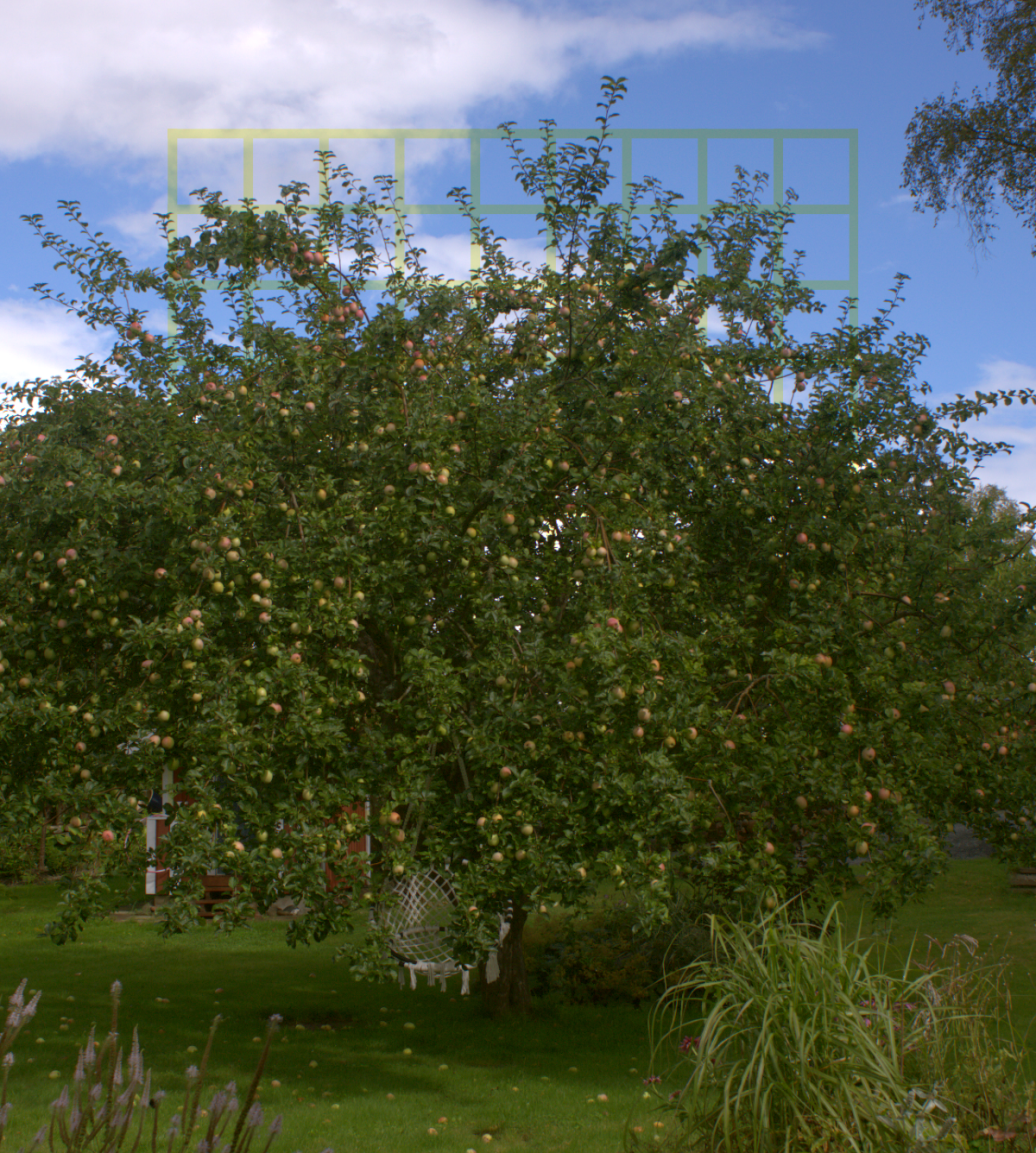
Illustration of the effect of background variation on the visibility of a window marking. A 30% blue-absorbing grid was digitally superimposed on the image. The reduction in human blue cone stimulation is comparable to what the 50% treatment in this study should have on the UV cones of a UVS bird. Against a blue rich and homogenous background (the sky) the grid becomes clearly visible but the introduced chromatic contrast is marginalised against a heterogenous and longwave dominated clutter (the apple tree). Photo by Olle Håstad.

It appears that the background against which ultraviolet markings are viewed strongly affects their visibility. The colour contrast introduced by manipulating ultraviolet content may be visible against backgrounds with little spatial variation in colour, such as clear sky and clouds. However, against highly variable backgrounds with low ultraviolet content, such as vegetation, the contrasts introduced by ultraviolet markings will not likely alert the bird to the presence of an obstacle (Fig. 10). Only to the UVS system and with at least a 50% UV reduction on perfect glass or 25% of real window reflectance was the contrast affected to such a degree that the marking could be detected against a background of a tree.

We used peafowl as our model VS species because it is the VS bird with the best known visual physiology (Hart, 2002). It is clearly ultraviolet sensitive, thanks to its fairly UV-transmissive ocular media, and this makes our comparisons to UVS birds conservative. Indeed some VS species can have even less ultraviolet absorbing ocular media than that of the peafowl, such as shown in bowerbirds (Coyle et al., 2012). Conversely, many species have more ultraviolet absorbing media (Lind et al., 2014). Light contrast introduced by ultraviolet window markings may be invisible to these birds because the signal is absorbed before reaching the retina. In raptors for example, the VS species of perhaps the highest concern, ultraviolet vision may be more limited than in our model species, as shown by their relatively ultraviolet absorbing ocular media (Lind et al., 2013).

**Figure 11:**
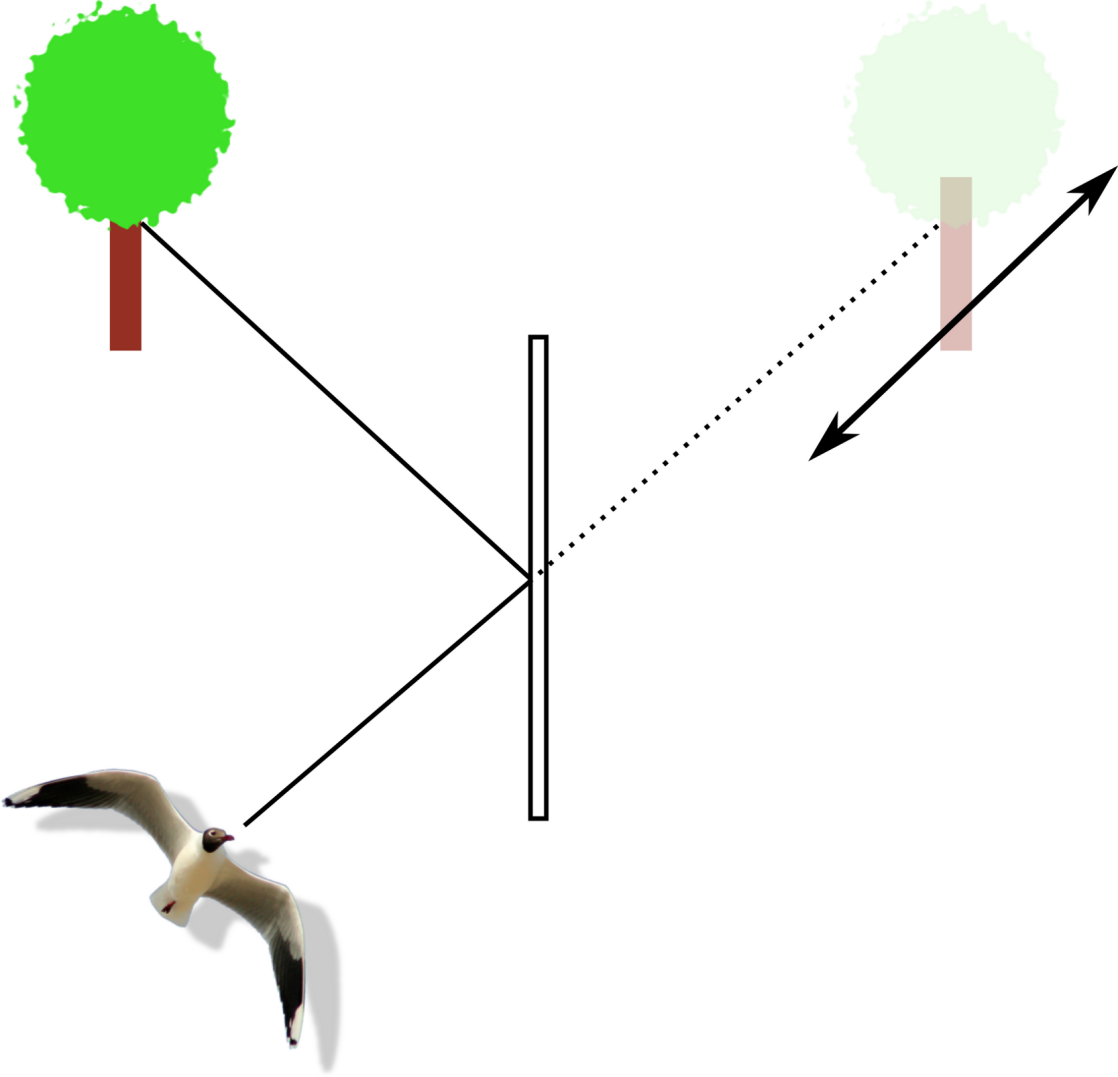
Depiction of the focal range around an object of interest reflected in a window. Reflection (solid lines) makes the object of interest (the tree) appear to be located behind the window (along the dotted line). The bird focusses on the tree, which is far enough from the window relative to the distance between the bird and the window that anything on the glass itself falls out of the focal range (depth of field: double headed arrow).

The window markings in this study were simulated as abrupt changes in intensity of certain wavelengths. That way they would be perceived as sharp boundaries of chromatic contrast in the natural scene. In practical applications however real ultraviolet markings will be perceived as having soft boundaries and therefore be less likely to catch the attention of birds. One reason is that spatial resolution is relatively low in most bird species, compared to humans (reviewed in Lind et al., 2012). Another is that birds are more likely to focus on items in a reflected or transmitted scene, such as a food source or a landing site, than on the glass surface in their flight path. Window markings will be more or less out of focus and therefore lower in perceived contrast the closer the bird comes to the window (Fig. 11). As the depth of field in the optics of a bird’s eyes decreases with eye size, this out of focus effect is more likely to be a problem to large birds than to small passerines.

In this study we have considered all chromatic contrasts to have equal value to object detection. This may not be true as the general assumption is that the double cones form the primary system for object recognition in birds, both when stationary (e.g., Jones & Osorio, 2004) and moving (von Campenhausen & Kirschfeld, 1998), and the double cones’ sensitivity lies outside the ultraviolet range, mainly located in the longer wavelengths (e.g., Hart & Hunt, 2007). Ultraviolet markings will not effectively alert birds to the presence of obstacles in their flight path if signals from the UV cones do not add into temporal resolution (Rössler, Laube & Weihs, 2007; Haupt, 2011) or spatial resolution, and then the chromatic contrasts considered in this study would be practically irrelevant to the bird-window collision problem. Data clarifying the role of single versus double cones in visual perception are however scarce. There is no direct evidence that spatial or temporal perception excludes the UV cone but one study does suggest a UV cone contribution to temporal resolution (Rubene et al., 2010).

## Conclusions

Our purpose with this study was to test the visibility of ultraviolet window markings under perfect and ideal circumstances, to evaluate whether it is at all possible to make window glass visible to birds without changing its appearance to humans. We conclude that ultraviolet markings may prevent window collisions in birds, but only for those with UVS vision, such as gulls, parrots and Passerida passerines and not for VS species, to which most other birds belong.
